# Inhalation of specific anti-*Pseudomonas aeruginosa* IgY antibodies transiently decreases *P. aeruginosa* colonization of the airway in mechanically ventilated piglets

**DOI:** 10.1186/s40635-019-0246-1

**Published:** 2019-04-08

**Authors:** A. Otterbeck, K. Hanslin, E. Lidberg Lantz, A. Larsson, J. Stålberg, M. Lipcsey

**Affiliations:** 1 Anesthesiology and Intensive Care, Department of Surgical Sciences, Uppsala University, Uppsala, Sweden; 2 Section of Clinical Chemistry, Department of Medical Sciences, Uppsala University, Uppsala, Sweden; 3 Hedenstierna laboratory, CIRRUS, Anesthesiology and Intensive Care, Department of Surgical Sciences, Uppsala University, Uppsala, Sweden

**Keywords:** Pneumonia, Nosocomial, VAP, HAP

## Abstract

**Background:**

*P. aeruginosa* is a pathogen frequently resistant to antibiotics and a common cause of ventilator-associated pneumonia (VAP). Non-antibiotic strategies to prevent or treat VAP are therefore of major interest. Specific polyclonal avian IgY antibodies have previously been shown to be effective against pneumonia caused by *P. aeruginosa* in rodents and against *P. aeruginosa* airway colonization in patients.

**Objectives:**

To study the effect of specific polyclonal anti-*P. aeruginosa* IgY antibodies (*Pa-*IgY) on colonization of the airways in a porcine model.

**Method:**

The pigs were anesthetized, mechanically ventilated, and subject to invasive hemodynamic monitoring and allocated to either receive 10^9^ CFU nebulized *P. aeruginosa* (control, *n* = 6) or 10^9^ CFU nebulized *P. aeruginosa* + 200 mg *Pa-*IgY antibodies (intervention, *n* = 6). Physiological measurement, blood samples, and tracheal cultures were then secured regularly for 27 h, after which the pigs were sacrificed and lung biopsies were cultured.

**Results:**

After nebulization, tracheal growth of *P. aeruginosa* increased in both groups during the experiment, but with lower growth in the *Pa-*IgY-treated group during the experiment (*p* = 0.02). Tracheal growth was 4.6 × 10^3^ (9.1 × 10^2^–3.1 × 10^4^) vs. 4.8 × 10^4^ (7.5 × 10^3^–1.4 × 10^5^) CFU/mL in the intervention group vs. the control group at 1 h and 5.0 × 10^0^ (0.0 × 10^0^–3.8 × 10^2^) vs. 3.3 × 10^4^ (8.0 × 10^3^–1.4 × 10^5^) CFU/mL at 12 h in the same groups. During this time, growth in the intervention vs. control group was one to two orders of ten lower. After 12 h, the treatment effect disappeared and bacterial growth increased in both groups. The intervention group had lower body temperature and cardiac index and higher static compliance compared to the control group.

**Conclusion:**

In this porcine model, *Pa*-IgY antibodies lessen bacterial colonization of the airways.

**Electronic supplementary material:**

The online version of this article (10.1186/s40635-019-0246-1) contains supplementary material, which is available to authorized users.

## Background

*Pseudomonas aeruginosa* is an opportunistic pathogen in humans known to host several antibiotic resistance mechanisms [1, 2]. The most lethal infections caused by *P. aeruginosa* are pneumonias of which ventilator-associated pneumonia (VAP) is a common clinical phenotype [3, 4]. VAP is believed to occur as bacteria-containing fluids from the oropharynx leak into and colonize the lower airways, the natural defense mechanisms being attenuated with sedation and the use of an endotracheal tube on which biofilm formation might also contribute [5, 6]. *P. aeruginosa* is a common pathogen in VAP, being responsible for about one in five cases in parts of the world [1].

IgY antibodies are large (167,250 kDa) monomeric antibodies produced in birds and reptiles with a molecular weight resembling mammalian IgG. The ability to harvest IgY from eggs laid by hens inoculated with *P. aeruginosa* provides a cheap and effective production method of specific polyclonal anti-*P. aeruginosa* IgY antibodies (*Pa-*IgY) [7]. These antibodies bind primarily to the flagella of these bacteria and facilitate opsonization by augmenting the phagocytic activity of polymorphonuclear neutrophils [8]. Two studies have shown that *Pa*-IgY can increase the time to airway colonization by *P. aeruginosa* in patients with cystic fibrosis [9, 10]. Another study administered *Pa-*IgY in a murine pneumonia model and found mainly prophylactic properties but also reduced pulmonary bacterial load with post-exposure treatment [11]. No study has previously investigated the prophylactic properties of *Pa-*IgY in a model of VAP development.

Today, the foundation of treating VAP is the effective use of antibiotics, something that is increasingly difficult with the increasing presence of resistant bacteria. To maintain the possibility of treating VAP caused by *P. aeruginosa* before resistance to available treatments becomes too widespread, novel strategies have to be examined. The objective of this study was to examine if it is possible to prevent initial airway colonization by *P. aeruginosa* with the use of *Pa*-IgY in an animal model with anesthetized and mechanically ventilated piglets. We used a model with nebulized delivery of *P. aeruginosa* predominantly to the large airways resulting in tracheobronchial colonization [12]. Our hypothesis was that the administration of *Pa*-IgY decreases the colonization by *P. aeruginosa* of the airways. The primary endpoint of this study is the growth of *P. aeruginosa* in tracheal cultures over time.

## Materials and methods

### Ethical approval

Ethical approval was received from the local animal ethics committee (application C155/14), and all animals were handled according to guidelines from the Animal Ethics Board (Uppsala, Sweden) and the European Union’s directives for animal research.

### Study protocol

This study was a prospective experimental animal study. Twelve crossbred Norwegian landrace pigs, 6–8 weeks old, were used. The experiments were carried out in an animal research facility at Uppsala University with experienced staff and in an ICU-like setting. All pigs were sedated with tiletamine/zolazepam 6 mg/kg (Zoletil, Virbac, Stockholm, Sweden) and xylazine 2.2 mg/kg (Rompun, Bayer, Köpenhamn, Denmark). Anesthesia was induced with 100 mg ketamine (Ketaminol, Intervet, Stockholm, Sweden) and 20 mg morphine (Morfin Meda, Meda, Solna, Sweden) and maintained with 1 g pentobarbital (Pentobarbitalnatrium, Apoteket, Kungens Kurva, Sweden) and 32.5 mg morphine mixed in 1000 mL of 25 mg/mL glucose given at 8 mL/kg/h. Relaxation was achieved using rocuronium 10 mg/mL (Esmeron, MSD, Stockholm, Sweden) infused at 2.5 mg/kg/h. Initial fluid was given as a bolus of 20 mL/kg Ringer’s acetate (Ringer-acetat, Fresenius Kabi, Uppsala, Sweden), and the same fluid was given as maintenance at 2 mL/kg/h intravenously. All pigs were mechanically ventilated (ratio of inspiratory to expiratory time 1:2, fraction inspired oxygen (FiO_2_) 0.3, tidal volume 10 mL/kg, respiratory rate 25, positive end-expiratory pressure (PEEP) 5 cmH_2_O) through a tracheostomy and received a central venous catheter, a pulmonary artery catheter, an arterial catheter, and a suprapubic urinary catheter. After preparation, the experiment started and carried on for 27 h with regular data collection. The experiment was carried out with animals lying on their side, changing side every 6 h followed by alveolar recruitment.

Additional morphine and ketamine was administered as needed to keep the animals anesthetized. Noradrenaline 20 μg/mL (Noradrenalin, Hospira Nordic, Stockholm, Sweden) was administered as a continuous infusion starting at 5 mL/h and increased as needed to maintain mean arterial pressure (MAP) > 60 mmHg. At cardiac output < 2 L/min, a clinical decision was made to either increase the rate of noradrenaline or to give a 15 mL/kg bolus of Ringer’s acetate. Normoventilation (PaCO_2_ 4.5–6.5 kPa) was achieved by adjusting tidal volume. Oxygenation target (PaO_2_ 10–30 kPa) was achieved by adjusting FiO_2,_ and for repeated hypoxemia, PEEP was incrementally increased. The animals were heated as necessary using heating pads, fluid warmers, and covers to maintain a body temperature between 35 and 42 °C. At the end of the experiment, the pigs were sacrificed using 20 mL KCl.

### IgY production

The method used for the production of *Pa*-IgY has been previously described [13, 14]. Briefly, White Leghorn hens were injected intramuscularly with inactivated *P. aeruginosa* (and Freund’s incomplete adjuvant), and identical booster injections were administered at 4-week intervals. After the second booster injection, the eggs were collected and the yolks were isolated. Polyethylene glycol (PEG) was added to the yolks twice in a precipitation process to isolate the yolk proteins. Ammonium sulfate was then added to remove the PEG and further purify large yolk proteins. This achieves a *Pa*-IgY fraction with a purity of more than 90%. The activity of purified *Pa*-IgY was measured with ELISA to yield equally active batches resulting in concentrations of *Pa-*IgY at approximately 5 mg/mL as measured spectrophotometrically. *Pa*-IgY was donated by Immunsystem I. M. S. AB (Uppsala, Sweden).

### Intervention

The pigs were allocated randomly to either receive *Pa-*IgY (intervention, *n* = 6) and *P. aeruginosa*, *P. aeruginosa* only (control, *n* = 6), *Pa*-IgY only (*n* = 3,) or anesthesia only (*n* = 3). The last two groups were only included as controls to detect effects of *Pa*-IgY and anesthesia, and their data are not included in the main analysis and can be found in (Additional file 1: Table S1A-7) together with a flowchart of the study groups (Additional file 1: Figure A1). *Pa*-IgY was administered immediately before the administration of *P. aeruginosa.* Ten milliliters of 5 mg/mL *Pa*-IgY was administered through a jet nebulizer at a flow of 6 L/min connected to the ventilatory circuit between the endotracheal tube and the Y-connection; this allowed for 10 mL of fluid to be nebulized during 10 min. Ten milliliters of 10^8^ CFU/mL *P. aeruginosa* (PA-103, ATC 29260, CCUG31589) in log phase was then administered with the same method of nebulization; the strain is a virulent clinical isolate from sputum with a type 3 secretion system and exotoxin A production. The strain has been tested for when log phase occurs (105 min) and is resistant to pig serum. Nebulizations were administered in the supine position before laying the pigs on their side.

### Data collection

Physiological parameters were measured regularly at predefined time points. Blood samples were collected in EDTA tubes and lithium heparin tubes every 3 h at 0–12 h and 24–27 h. These tubes were analyzed at the central laboratory of the hospital for complete blood counts, plasma cytokines, and plasma and urine creatinine levels. Blood gases were analyzed in blood gas analyzers at the animal research facility (ABL835- Flex Radiometer and OSM3 Oximeter Radiometer). Urine was collected during three time intervals, 0–12 h, 12–24 h, and 24–27 h to measure urine output and for calculation of creatinine clearance. Tracheal secretions were acquired through a 10 mL tracheal lavage followed by aspiration. One hundred microliters of the lavage was then serially diluted to determine the number of *P. aeruginosa* colony-forming units. Each dilution was cultured on a CLED plate. Blood cultures were drawn from the arterial catheter and 100 μL of blood was cultured on CLED plates. All cultures were incubated for at least 24 h at 37 °C. The colonies were then manually counted, and the most diluted culture with a growth of 30–300 colonies was recorded as the bacterial growth for that sample. Cultures were also acquired from a lower right lobe lung biopsy measuring 1 g which was minced, diluted in 200 μL NaCl, and then cultured on CLED culture plates.

### Statistical analysis

Data was tested for normality and is presented accordingly as mean ± SEM or median (IQR). Differences in baseline characteristics were tested for using an independent *t* test or Mann-Whitney *U* test according to normality. Culture results, cytokines, and arterial lactate levels were log-normally distributed and were thus log-transformed. To test differences for repeated measurements between groups and over time, mixed linear models (ANOVA III) were used. *p* < 0.05 was considered statistically significant. *Pa*-IgY and anesthesia only groups were not included in the statistical analysis.

## Results

### Baseline characteristics

The intervention group was similar to the control group at baseline (Table 1). Pigs in the intervention group weighed more than in the control group.

**Table 1:** Characteristics at baseline

Baseline characteristics		
	Intervention	Control
Weight (kg)*	31.2 ± 1.1	27.9 ± 0.73
HR (min^−1^)	98 ± 4	92 ± 6
MAP (mmHg)	81 ± 5	78 ± 5
CI (L/min/m^2^)	2.18 (0.72)	2.83 (0.62)
Arterial lactate (mmol/L)	2.2 ± 0.4	1.9 ± 0.2
Hemoglobin (g/L)	87 ± 1	87 ± 3
WBC (10^9^/L)	18.0 ± 2.7	15.6 ± 1.8
Neutrophil count (10^9^/L)	8.6 ± 2.1	7.0 ± 1.3
Core temperature (°C)	39.3 ± 0.2	39.5 ± 0.2
IL-6 (pg/mL)	31.0 ± 28.5	39.7 ± 20.2
TNF-α (pg/mL)	184.6 ± 16.9	166.6 ± 22.0
Static compliance (mL/cmH_2_O)	29.8 ± 3.9	29.3 ± 2.7
PaO_2_/FiO_2_ ratio (mmHg)	370 (34)	420 (56)
Plasma creatinine (μmol/L)	43 ± 2	50 ± 4

Values are mean ± SEM or median (IQR). *p < 0.05, tested according to normality with independent *t* test or Mann-Whitney *U* test. *HR* heart rate, *MAP* mean arterial pressure, *CI* cardiac index, *aLactate* arterial lactate, *WBC* white blood cell count, *IL-6* interleukin-6, *TNF-α* tumor necrosis factor α

### Physiological parameters and laboratory analysis (Fig. 1)

HR changed over time (*p* = 0.024) with no difference between groups. There was no difference in MAP between groups, and there was no difference in the total administered noradrenaline between groups, 137 μg (1705) in the control group vs 0 μg (4463) in the intervention group. CI increased over time in both groups (*p* = 0.001) with higher levels in the intervention group (*p* = 0.009). aLactate decreased over time in both groups with no difference between groups. There was no difference over time or between groups in Hb or WBC. Neutrophil granulocyte count (NGC) changed over time (*p* = 0.040) with no difference between groups. Core temperature changed over time in both groups (*p* < 0.001) with higher temperatures in the control group compared to the intervention group (0.027). IL-6 increased over time with no difference between groups (*p* < 0.001). There was no difference over time or between groups for TNF-α. Static compliance was lower in the control group compared to the intervention group (*p* = 0.039) during the experiment. PaO_2_/FiO_2_ ratio decreased over time in both groups (*p* = 0.021) with no difference between groups. There was no difference in creatinine clearance between groups or over time. The *Pa*-IgY only and anesthesia only groups had numerically lower HR, CI, and core temperature (see Additional file 1 for values). MAP and Hb were numerically lower in these two groups. The anesthesia only group had numerically higher static compliance and PaO_2_/FiO_2_ ratio.

**Fig. 1:**
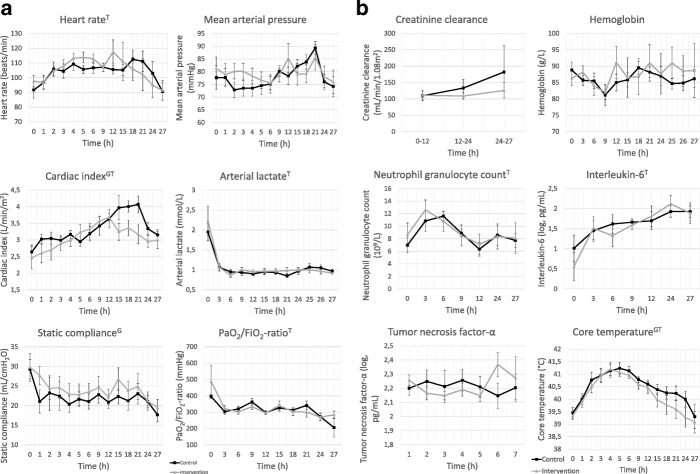
**a** Graphs representing physiological parameters and laboratory analysis over time. Data points represent mean and error bars represent SEM. Black line represents the control group and gray line represents the intervention group. G, significant group difference; T, significant difference over time. No parameters had a significant group-time interaction. **b** Graphs representing physiological parameters and laboratory analysis over time. Data points represent mean and error bars represent SEM. Black line represents the control group and gray line represents the intervention group. G, significant group difference; T, significant difference over time. No parameters had a significant group-time interaction

### Bacterial cultures

**Fig. 2:**
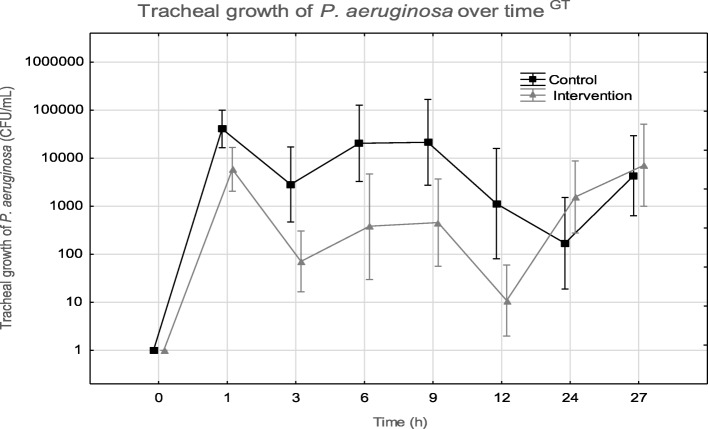
Tracheal growth of *P. aeruginosa* over time. Data points represent mean and error bars represent SEM. Black line represents the control group and gray line represents the intervention group. G, significant group difference; T, significant difference over time

There was an increase in tracheal growth of *P. aeruginosa* over time (*p* = 0.001) in all pigs with less growth in the intervention group compared to the control group (*p* = 0.02) (Fig. 2, Additional file 1: Table S1B for individual data). The bacterial growth was one to two orders of magnitude lower in the intervention group until 12 h. After 12 h, growth increased in both groups and the numerical difference between groups disappeared. Three pigs in the intervention group showed cultures with no growth of *P. aeruginosa* at various time points 0–12 h with one pig where *P. aeruginosa* was eradicated 3–27 h. In the control group, one pig had one culture without growth between 0 and 12 h and two pigs at 12 h. Pulmonary biopsies showed growth in only one pig from each group, 190 CFU/mL in the control group vs. 126 CFU/mL in the intervention group. There was no growth in blood cultures. The *Pa*-IgY only and anesthesia only groups had no growth of *P. aeruginosa* in cultures. The growth of endogenous flora (*Bordetella bronchiseptica*) at 0 h was seen in two pigs in the intervention group; *B. bronchiseptica* did not grow in any subsequent cultures. The growth of *E. coli* occurred in one pig from the control group and one pig from the intervention group at 24 h with *E. coli* with continued growth at 27 h in the pig from the control group.

## Discussion

In this study, we used a porcine model of large airway colonization by *P. aeruginosa* to investigate if a pre-exposure inhalation of *Pa-*IgY could reduce subsequent colonization by *P. aeruginosa.* Our results show that treatment with *Pa*-IgY reduces the growth of *P. aeruginosa* in tracheal cultures.

These data are in line with previous research on patients with cystic fibrosis where gargling of *Pa-*IgY increased time to initial colonization by *P. aeruginosa* [9, 10] It is also in line with studies on mice where prophylactic properties of *Pa*-IgY were found [11]. Yet, unlike the findings in that study, we saw a decreased effect of *Pa*-IgY after 12 h. Given the findings in mice, it is less likely that the loss of treatment effect after 12 h in this study is due to degradation of the IgY molecule. There is also evidence that the half-life of IgY in pig serum is closer to 48 h rather than 12 h [15]. Rather, this may be due to the fact that at the dose given there are unaffected bacteria that keep dividing, and eventually, the bacterial load is too large compared to the available *Pa*-IgY binding capacity for the treatment effect to persist. This could be mitigated by larger or repeated doses of *Pa-*IgY. Finally, theoretically emergence of bacterial resistance could also explain the decreased antibacterial effect of *Pa-*IgY. However, an in vivo study has shown that numbers of *P. aeruginosa* do not increase until 24 h after antimicrobial challenge [16]. One may speculate that given the short time to decreasing effect (12 h) and that the antibodies we use are polyclonal with several binding sites to the bacteria [8], bacterial resistance to *Pa*-IgY is less likely.

In our experiments, static compliance was higher in the intervention group than the control group. Although the mechanisms of this observation are not elucidated by our data, lower *P. aeruginosa* burden in *Pa*-IgY-treated animals could have contributed to better respiratory mechanics in this group. Core temperature was higher in the control group, which might imply an increased inflammatory response without *Pa-*IgY when the flagella of these bacteria can stimulate the immune system primarily via the TLR-5 receptors [17]. CI was also higher in the control group signifying an increased stress response. These findings in the control group are unspecific but could represent the initial signs of an infection. This is biologically plausible since colonization precedes infection and there is less colonization in the intervention group, delaying the onset of infection. The inflammatory basis of the difference in temperature and CI are contradicted by the lack of group difference in WBC, NGC, IL-6, or TNF-α. On the other hand, although the TLR-5 pathway induces several inflammatory mediators, including TNF-α and IL-6, its main effect is mediated through IL-8 which was not measured in this study [18].

This is as far as we know the first study to report the effect of *Pa*-IgY on lower respiratory tract colonization by *P. aeruginosa.* The strength of this study lies in its use of a mechanically ventilated large animal model in an ICU setting, resembling clinical VAP and allowing complex physiological measurements and repeated blood and tracheal sampling. The animals were also followed for more than 24 h, allowing the assessment of the length of *Pa*-IgY effect. The relatively small number of animals used is a limitation. However, these experiments are cumbersome costly and limitation of the number of animals is strongly encouraged by the ethical rules. Given the set alpha level, the number of animals impacts mainly type II error. Another limitation lies in the fact that the model only investigates *P. aeruginosa* colonization, not if *Pa*-IgY can prevent VAP by decreasing colonization, since this is not a VAP model. The nebulization of *P. aeruginosa* and *Pa*-IgY probably delivers a lower dose to the pig lung than what is nebulized due to losses in the ventilator circuit [19]. This has been accounted for by choosing larger doses of both nebulizations. The growth of *P. aeruginosa* and the treatment effect seen with *Pa*-IgY confirms effective nebulization. Also, in this study, we did not do any histopathological analysis of the lungs, since our main focus was bacterial growth. We did not take pulmonary biopsies from all lobes, as the pulmonary biopsies were taken from a standardized location in the right lower lobe to represent alveoli and bronchiole. However, we examined both lungs macroscopically, and this standardized biopsy location gave representative samples. We observed the pigs for more than a day. However, longer experiments could be of interest, especially for studying VAP, since bacterial growth may increase up to 72 h in the lungs [20]. Finally the study, although randomized, was not blinded; however, interventions allowed were performed to a preset protocol.

Future research in this area should explore the effect of *Pa-*IgY in both the prevention and post-exposure treatment of VAP. Also, studies on IgY against other pathogens than *P. aeruginosa* would be of interest. An earlier study has tested monoclonal antibodies for pneumonia caused by *Staphylococcus aureus* suggesting that antibodies are an attractive target for future human research [21]. If *Pa*-IgY is proven to be effective against *P. aeruginosa*, this can have great implications for patients even outside of VAP and should be studied accordingly. Since *P. aeruginosa* is an opportunistic pathogen, all immunocompromised patients could benefit from prevention and eventual treatment to spare both antibiotic use and suffering for the patient.

## Conclusions

In summary, in an anesthetized and mechanically ventilated porcine model, specific polyclonal anti-*P. aeruginosa* IgY antibodies can be used as prophylaxis to decrease colonization in the lower respiratory tract by *P. aeruginosa.* These findings are an important step towards new therapies for VAP in the race against antimicrobial resistance.

## Additional file

Additional file 1: **Table S1A-7**. Data stratified per group for complementary experimental groups. All data is measured according to what is described in the methods section. Number are mean ± SEM. **Figure S1A.** Flowchart of experimental groups. A flowchart describing the different experimental groups. **Table S1B**. Individual tracheal growth of *P. aeruginosa* stratified per group. Individual data of Tracheal growth of *P. aeruginosa* over time per pig (colony forming unit, CFU/mL). Data presented is growth in tracheal cultures at each time point during the experiment for each animal. CFU: colony forming unit. (DOCX 28 kb)

## Abbreviations

aLactate: Arterial lactate
CFU: Colony forming units
CI: Cardiac index
FiO_2_: Fraction of inspired oxygen
Hb: Hemoglobin
HR: Heart rate
IL-6: Interleukin-6
MAP: Mean arterial pressure
NGC: Neutrophil granulocyte count
*Pa-*IgY: Specific polyclonal anti-*P. aeruginosa* IgY antibodies
PEEP: Positive end-expiratory pressure
PEG: Polyethylene glycol
TNF-α: Tumor necrosis factor alpha
VAP: Ventilator-associated pneumonia
WBC: White blood cell count
