# Correction to: Inhalation of specific anti-Pseudomonas aeruginosa IgY antibodies transiently decreases *P. aeruginosa* colonization of the airway in mechanically ventilated piglets

**DOI:** 10.1186/s40635-019-0253-2

**Published:** 2019-05-09

**Authors:** A. Otterbeck, K. Hanslin, E. Lidberg Lantz, A. Larsson, J. Stålberg, M. Lipcsey

**Affiliations:** 1 Anesthesiology and Intensive Care, Department of Surgical Sciences, Uppsala University, Uppsala, Sweden; 2 Section of Clinical Chemistry, Department of Medical Sciences, Uppsala University, Uppsala, Sweden; 3 Hedenstierna laboratory, CIRRUS, Anesthesiology and Intensive Care, Department of Surgical Sciences, Uppsala University, Uppsala, Sweden

**Correction to: Intensive Care Med Exp**

**https://doi.org/10.1186/s40635-019-0246-1**

Following publication of the original article [1], the authors flagged that an incorrect piece of data is given in the *Materials and Methods* section of the article.

In this section it is stated “Ten milliliters of 10^8^ CFU/mL *P. aeruginosa*”.

Please note that this should read: “Ten milliliters of 10^9^ CFU/mL *P. aeruginosa*”.

The authors apologize for this error.
