# Supplementary material for: Inhalation of specific anti-Pseudomonas aeruginosa IgY antibodies transiently decreases P. aeruginosa colonization of the airway in mechanically ventilated piglets
Source: Intensive Care Med Exp. 2019 Apr 8;7:21. doi: 10.1186/s40635-019-0246-1 (PMC6453987; doi:10.1186/s40635-019-0246-1)
Supplement: Supplementary file 1 — Table S1A-7. Data stratified per group for complementary experimental groups. All data is measured according to what is described in the methods section. Number are mean ± SEM. Figure S1A. Flowchart of experimental groups. A flowchart describing the different experimental groups. Table S1B. Individual tracheal growth of P. aeruginosa stratified per group. Individual data of Tracheal growth of P. aeruginosa over time per pig (colony forming unit, CFU/mL). Data presented is growth in tracheal cultures at each time point during the experiment for each animal. CFU: colony forming unit. (DOCX 28 kb) [file 40635_2019_246_MOESM1_ESM.docx]

**Supplemental data**

The supplemental data contains:

- Measurements on the *Pa*-IgY only and anesthesia only groups (Table A1-A7). All data presented are mean ± SEM.
- A flowchart describing the different experimental groups.
- A table of tracheal growth of *P. aeruginosa* of the individual pigs stratified per group.

**Table A. Data stratified per group for complementary experimental groups**

| \| **1. Heart rate (beats/min)** \| \| \| \| --- \| --- \| --- \| \|  \|  \|  \| \|  \| *Pa*-IgY only \| Anesthesia only \| \| 0h \| 99±8 \| 102±8 \| \| 1h \| 85±2 \| 70±5 \| \| 2h \| 84±2 \| 80±12 \| \| 3h \| 83±5 \| 81±11 \| \| 4h \| 83±6 \| 89±7 \| \| 5h \| 92±5 \| 89±9 \| \| 6h \| 96±1 \| 87±9 \| \| 9h \| 96±5 \| 92±4 \| \| 12h \| 98±4 \| 100±8 \| \| 15h \| 99±6 \| 93±9 \| \| 18h \| 87±9 \| 96±8 \| \| 21h \| 102±11 \| 85±12 \| \| 24h \| 95±10 \| 96±11 \| \| 27h \| 93±9 \| 98±12 \| |
| --- | --- | --- | --- | --- | --- | --- | --- | --- | --- | --- | --- | --- | --- | --- | --- | --- | --- | --- | --- | --- | --- | --- | --- | --- | --- | --- | --- | --- | --- | --- | --- | --- | --- | --- | --- | --- | --- | --- | --- | --- | --- | --- | --- | --- | --- | --- | --- | --- | --- | --- | --- |

| **2. Cardiac index (L/min/m^2^)** | | |
| --- | --- | --- |
|  |  |  |
|  | *Pa*-IgY only | Anesthesia only |
| 0h | 3.1±0.5 | 3.6±0.4 |
| 1h | 2.3±0.3 | 2.9±0.5 |
| 2h | 2.4±0.3 | 2.7±0.3 |
| 3h | 2.4±0.2 | 2.5±0.2 |
| 4h | 2.5±0.2 | 2.6±0.3 |
| 5h | 2.5±0.2 | 2.7±0.2 |
| 6h | 2.6±0.2 | 2.5±0.2 |
| 9h | 2.7±0.2 | 2.6±0.1 |
| 12h | 3.7±0.6 | 3.3±0.5 |
| 15h | 3.4±0.4 | 2.9±0.4 |
| 18h | 3.2±0.6 | 2.8±0.1 |
| 21h | 3.2±0.3 | 2.7±0.1 |
| 24h | 3.9±0.7 | 3.2±0.6 |
| 27h | 3.2±0.3 | 3.9±1.0 |

| **3. Mean arterial pressure (mmHg)** | | |
| --- | --- | --- |
|  |  |  |
|  | *Pa*-IgY only | Anesthesia only |
| 0h | 73±3 | 82±3 |
| 1h | 69±5 | 76±3 |
| 2h | 69±7 | 78±5 |
| 3h | 69±7 | 80±9 |
| 4h | 73±5 | 85±6 |
| 5h | 74±6 | 83±5 |
| 6h | 74±7 | 88±7 |
| 9h | 77±5 | 78±4 |
| 12h | 76±5 | 75±2 |
| 15h | 89±5 | 83±1 |
| 18h | 79±8 | 83±5 |
| 21h | 78±2 | 89±6 |
| 24h | 91±4 | 85±7 |
| 27h | 89±4 | 88±11 |

| **4. Core temperature (°C)** | | |
| --- | --- | --- |
|  |  |  |
|  | *Pa*-IgY only | Anesthesia only |
| 0h | 39.0±0.6 | 38.1±0.4 |
| 1h | 39.4±0.7 | 38.3±0.5 |
| 2h | 39.6±0.8 | 38.6±0.5 |
| 3h | 39.6±0.8 | 38.9±0.5 |
| 4h | 40.0±0.8 | 39.0±0.5 |
| 5h | 40.2±0.8 | 39.4±0.4 |
| 6h | 40.4±0.7 | 39.6±0.4 |
| 9h | 40.9±0.6 | 40.1±0.5 |
| 12h | 40.0±0.6 | 39.9±0.6 |
| 15h | 39.7±0.9 | 39.7±1.1 |
| 18h | 38.9±1.6 | 39.3±1.0 |
| 21h | 38.2±2.2 | 38.7±1.2 |
| 24h | 37.8±2.3 | 38.4±1.1 |
| 27h | 38.1±2.2 | 38.5±0.9 |

| **5. Hemoglobin (g/L)** | |  |
| --- | --- | --- |
|  |  |  |
|  | *Pa*-IgY only | Anesthesia only |
| 0h | 90±2 | 91±5 |
| 3h | 85±1 | 88±5 |
| 6h | 82±3 | 90±5 |
| 9h | 85±4 | 91±2 |
| 12h | 90±6 | 93±3 |
| 15h | 91±1 | 90±2 |
| 18h | 92±1 | 91±3 |
| 21h | 95±4 | 95±2 |
| 24h | 101±2 | 100±3 |
| 25h | 92±2 | 96±2 |
| 26h | 95±3 | 97±3 |
| 27h | 98±3 | 100±3 |

| **6. Static compliance (mL/cmH_2_O)** | | |
| --- | --- | --- |
|  |  |  |
|  | *Pa*-IgY only | Anesthesia only |
| 0h | 34±4 | 37±5 |
| 1h | 26±5 | 32±2 |
| 2h | 23±3 | 25±0 |
| 3h | 24±3 | 27±3 |
| 4h | 23±3 | 24±4 |
| 5h | 21±3 | 24±2 |
| 6h | 23±2 | 24±3 |
| 9h | 24±3 | 28±5 |
| 12h | 22±1 | 23±3 |
| 15h | 20±1 | 26±3 |
| 18h | 27±4 | 24±2 |
| 21h | 26±4 | 28±3 |
| 24h | 19±1 | 21±4 |
| 27h | 17±1 | 19±3 |

| **7. PaO_2_/FiO_2_-ratio (mmHg)** | | |
| --- | --- | --- |
|  |  |  |
|  | *Pa*-IgY only | Anesthesia only |
| 0h | 417±65 | 424±61 |
| 3h | 365±26 | 380±37 |
| 6h | 343±27 | 326±23 |
| 9h | 331±27 | 393±35 |
| 12h | 303±23 | 348±27 |
| 15h | 351±21 | 378±61 |
| 18h | 329±7 | 362±42 |
| 21h | 321±38 | 427±57 |
| 24h | 240±65 | 348±75 |
| 27h | 276±40 | 336±22 |

**Table A1-7.** Data stratified per group for complementary experimental groups. All data is measured according to what is described in the methods section. Number are mean ± SEM

**Fig A1. Flowchart of experimental groups**

**Figure A1.** A flowchart describing the different experimental groups

**Table B1. Individual tracheal growth of *P. aeruginosa* stratified per group**

| Animal |  |  |  |  |  |  |  |  |
| --- | --- | --- | --- | --- | --- | --- | --- | --- |
| Control | **0h** | **1h** | **3h** | **6h** | **9h** | **12h** | **24h** | **27h** |
| 1 | 0 | 1170000 | 1170000 | 6700 | 65000 | 34000 | 168000 | 168000 |
| 2 | 0 | 81000 | 6000 | 40000 | 450000 | 173000 | 0 | 580 |
| 3 | 0 | 160000 | 13700 | 2220 | 100 | 32000 | 30 | 24200 |
| 4 | 0 | 4000 | 10000 | 3500000 | 510000 | 330000 | 94000 | 330000 |
| 5 | 0 | 5000 | 1070 | 410 | 92000 | 0 | 50 | 8200 |
| 6 | 0 | 14800 | 0 | 84000 | 690 | 0 | 0 | 0 |
| Intervention |  |  |  |  |  |  |  |  |
| 7 | 0 | 6900 | 540 | 62000 | 1530 | 500 | 350000 | 2190 |
| 8 | 0 | 39000 | 70 | 0 | 140 | 0 | 4200 | 50000 |
| 9 | 0 | 2200 | 0 | 30 | 280 | 10 | 12000 | 860000 |
| 10 | 0 | 310000 | 5100 | 450000 | 48000 | 15100 | 1720 | 137000 |
| 11 | 0 | 480 | 660 | 3800 | 3100 | 0 | 470 | 10000 |
| 12 | 0 | 460 | 0 | 0 | 0 | 0 | 0 | 0 |
|  | | | | | | | | |

**Table B1:** Individual data of Tracheal growth of P. aeruginosa over time per pig (colony forming unit, CFU/mL). Data presented is growth in tracheal cultures at each time point during the experiment for each animal. CFU: colony forming unit.
